# Case Report: Calcium sulfate antibiotic beads and bone morphogenetic protein-2–loaded hydroxyapatite and allograft for the treatment of infected delayed union in a dog

**DOI:** 10.3389/fvets.2026.1710429

**Published:** 2026-03-04

**Authors:** Hyobum Cho, Byung-Jae Kang, Junhyung Kim

**Affiliations:** 1 College of Veterinary Medicine and Institute of Veterinary Science, Kangwon National University, Chuncheon-si, Republic of Korea; 2 Department of Veterinary Clinical Sciences, College of Veterinary Medicine and Research Institute for Veterinary Science, Seoul National University, Seoul, Republic of Korea

**Keywords:** bone morphogenetic protein-2, bone tissue engineering, calcium sulfate antibiotic beads, case report, delayed union, objective gait analysis, osteomyelitis, regenerative medicine

## Abstract

A 6-year-old male German Shepherd dog was presented with a closed, highly comminuted fracture of the left tibia caused by a fall. The fracture was stabilized using a plate-and-rod construct that was further protected with an external skeletal fixator. However, the surgery resulted in an external torsional deformity of the left tibia. Therefore, 23 weeks after the first surgery, an additional deformity-correction surgery was performed based on the tibial torsion angle measured from computed tomography scans. At 28 weeks after the first surgery, osteomyelitis at the osteotomy site in the left tibia, caused by methicillin-resistant *Staphylococcus aureus*, was diagnosed based on culture and antimicrobial susceptibility testing, and this infection was considered to hinder bone healing. At 28 weeks after the first surgery, a third surgery was performed in which calcium sulfate antibiotic beads and antibiotic-impregnated collagen sponges, together with hydroxyapatite and allografts loaded with recombinant human bone morphogenetic protein-2, were grafted for treatment of osteomyelitis and to promote bone healing. Six weeks following the application of antibiotic beads and bone graft materials, clinical bone union was observed. Complete bone healing was confirmed using radiographic imaging, and functional recovery was verified using objective gait analysis. The implant was subsequently removed to prevent stress shielding and the associated peri-implant bone loss. In conclusion, a tissue engineering strategy combining local antibiotic delivery using calcium sulfate antibiotic beads with bone graft substitutes loaded with recombinant human bone morphogenetic protein-2 can overcome the limitations of systemic antibiotic therapy and may be a viable option for treating infected tibial delayed union.

## 1 Introduction

Tibial fractures are predisposed to osteomyelitis and other healing complications because of limited craniomedial soft tissue coverage (1). In one study, delayed union occurred in 13.9% of fractures, and tibia–fibula fractures accounted for 9.2% of delayed unions (2). Based on this study, dogs with comminuted fractures have a significantly increased risk of healing complications, including delayed union and non-union, highlighting the importance of appropriate management for these fractures.

In a recent study of comminuted long-bone fractures, open fractures had a higher postoperative complication rate than closed fractures, and osteomyelitis was one of the most common complications, occurring in 6/79 cases (7.6%) (3). Standard therapies include systemic antibiotics, implant removal, and debridement (4), but efficacy of treatment may be limited by prolonged administration, owner compliance, poor vascularity, and biofilm (5). Local antibiotic delivery can achieve high concentrations while minimizing systemic adverse effects (6, 7). Although polymethylmethacrylate (PMMA) has been widely used as a local carrier, its exothermic curing reaction limits the use of heat-sensitive antibiotics and its nonbiodegradability often necessitates secondary removal surgery (6). Calcium sulfate is resorbable, sets with minimal temperature rise, allows incorporation of heat-sensitive antibiotics, and provides antibiotic elution patterns comparable to PMMA (7). Calcium sulfate antibiotic beads have been used in open fractures, chronic osteomyelitis, and periprosthetic joint infections (6, 8).

Infected delayed union or non-union is traditionally managed using a staged approach that includes aggressive surgical debridement of infected or necrotic bone, copious lavage with establishment of drainage, appropriate systemic antimicrobial therapy, and rigid stabilization, with cancellous bone grafting performed when the surgical bed is considered suitable or delayed until infection is controlled (9). Definitive stabilization following initial infection control has been reported using plate-and-screw fixation, circular external skeletal fixation (ESF) with Ilizarov-type constructs, or an interlocking intramedullary nail (9–11). When extensive debridement results in segmental bone loss, Ilizarov-based distraction osteogenesis and bone transport using circular fixation have been described for infected defects and for limb-sparing management of primary bone tumors (12, 13). This strategy is resource-intensive, may require multiple surgeries and prolonged treatment, and may not be feasible in all settings (12, 13). These limitations highlight the need for strategies that address infection and impaired bone healing concurrently. Evidence on single-stage management remains limited to case reports and small case series (14, 15). This case report describes the successful management of an infected delayed union using calcium sulfate antibiotic beads combined with recombinant human bone morphogenetic protein-2 (rhBMP-2)–loaded hydroxyapatite (HA) and allograft to provide local infection control and bone regeneration.

## 2 Case description

### 2.1 Patient information

**Figure 1:**
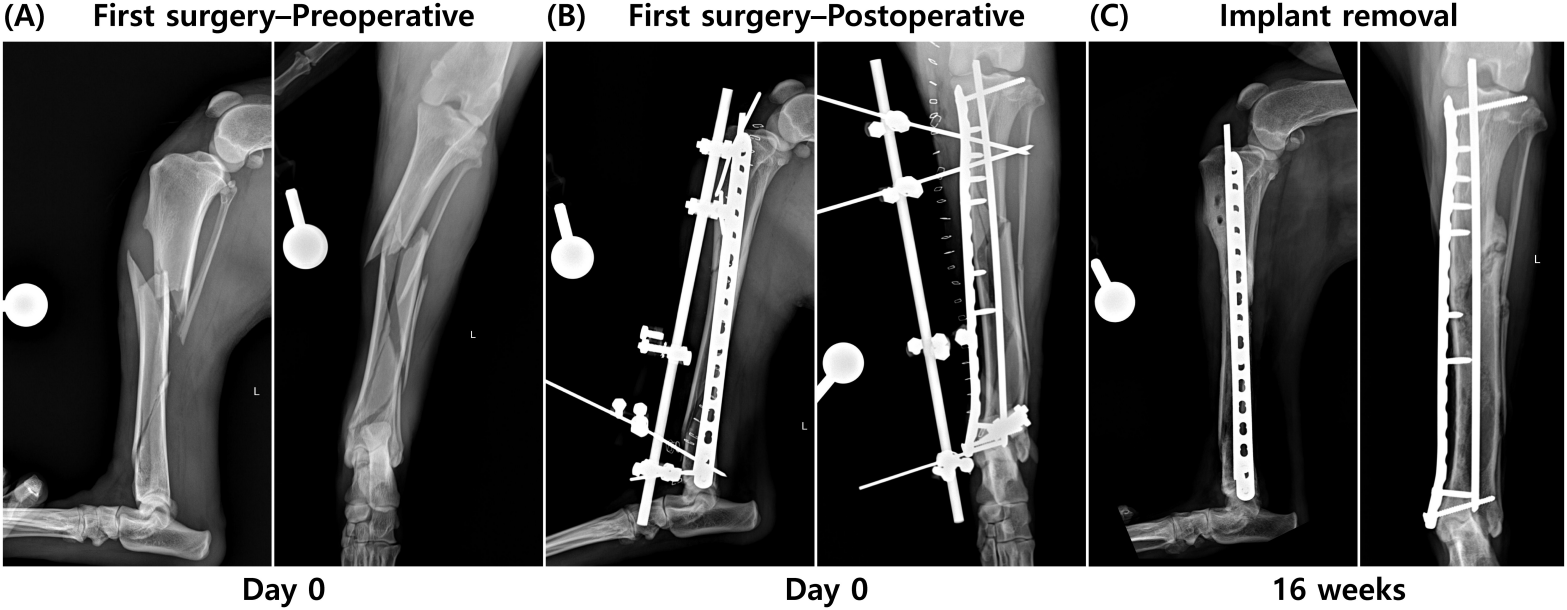
Radiographs obtained from the period before the first surgery to 16 weeks postoperatively. **(A)** A closed, multifragmentary diaphyseal fracture of the left tibia at initial presentation. **(B)** Postoperative radiograph obtained immediately after the first surgery, in which plate-rod stabilization and type 1 external skeletal fixation (ESF) were applied. **(C)** Radiograph taken 16 weeks postoperatively, after removal of the ESF, showing development of a hypertrophic non-union at the lateral aspect of the proximal one-third of the tibial diaphysis.

A 6-year-old male German Shepherd (35 kg) was presented after falling from 2 m with non-weight-bearing left hindlimb lameness and pain on palpation. No wounds consistent with an open fracture were identified. Left tibial radiographs showed a closed multifragmentary diaphyseal fracture with a spiral configuration of the main fragment, extending from the proximal third to the distal diaphysis (16) (Figure 1A). In accordance with institutional policy, informed consent was obtained from the owner to use patient data for educational and research purposes.

### 2.2 Surgical management and follow-up

Fracture stabilization was achieved using an Open-But-Do-Not-Touch (OBDNT) reduction technique, with plate-and-rod fixation, supported by a type I ESF. Under anesthesia, the patient was positioned in dorsal recumbency. A medial tibial approach with minimal dissection was used. Multiple fracture fragments were not amenable to anatomic reduction and were left in place without manipulation to reduce the risk of sequestrum formation. A 4.5 mm intramedullary (IM) pin was placed normograde under fluoroscopic guidance to restore alignment, followed by application of a 16-hole 3.5 mm locking compression plate (LCP; DePuy Synthes, Solothurn, Switzerland). The first screw proximally and the ninth screw distally were placed as bicortical non-locking screws, whereas the second through eighth screws were placed as monocortical locking screws. A type I ESF was placed on the craniomedial tibia (Figure 1B), and closure was performed.

At 3 weeks postoperatively, a draining tract developed at the distal part of the skin incision. Wound culture and susceptibility testing identified *Staphylococcus aureus*. Doxycycline was selected and administered at 10 mg/kg orally twice daily for 2 weeks. The infection subsequently resolved. At 16 weeks postoperatively, radiographs showed bridging callus across most fracture lines, with a persistent hypertrophic non-union laterally at the proximal one-third of the diaphysis. Because cortical thinning could suggest occult infection, the ESF was removed (Figure 1C). External tibial torsion became evident after ESF removal.

### 2.3 Deformity correction and follow-up

**Figure 2:**
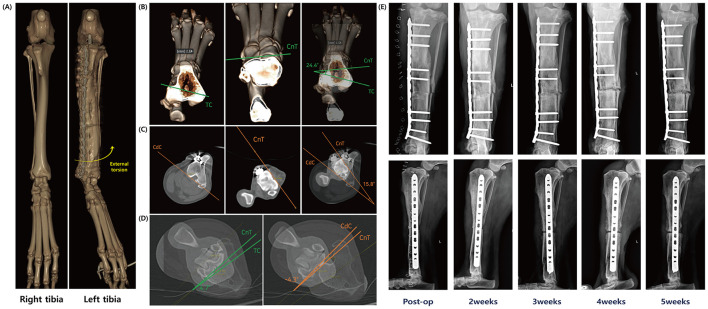
Process of torsional deformity measurement and surgical correction. **(A)** Three-dimensional reconstructed CT images of the normal limb (right tibia) and the deformed limb (left tibia). External torsion of the left tibia is evident. **(B, C)** Preoperative measurement of tibial torsional deformity: the tibial torsion angle was measured using two different methods. **(D)** Postoperative measurement of tibial torsional deformity: torsion was assessed using computed tomography with same methods. **(E)** Serial radiographs obtained immediately after torsional deformity correction and at 2, 3, 4, and 5 weeks postoperatively, arranged chronologically from left to right. Over time, these images demonstrate the development of non-union due to osteomyelitis following the deformity correction surgery.

A computed tomography (CT) scan of both hindlimbs was performed to assess the torsional deformity, which was confined to the transverse plane and showed no angular deformities in the sagittal and frontal planes (Figure 2A). The tibial torsion angle (TTA) was measured using the method described by Aper et al. (17). The angles between the transcondylar axis (TC) and the cranial tibial axis (CnT), and between the caudal condylar axis (CdC) and the cranial tibial axis (CnT), were 24.4° and 15.8°, respectively (Figures 2B–C). Based on these measurements, CT analysis indicated that a torsional correction of approximately 20° was required, corresponding to a linear correction of 6.3 mm medially at the osteotomy site, calculated using the formula described by Longo et al. (18).

Twenty-three weeks after the first surgery, a deformity-correction surgery (second surgery) was performed. Through a medial approach, the IM-pin and medial plate were removed. A tibial osteotomy was created at the distal two-thirds of the tibia, away from the original fracture site. The distal tibial segment was rotated to achieve alignment and stabilized with a 14-hole 3.5 mm LCP (DePuy Synthes, Solothurn, Switzerland) secured with bicortical 3.5 mm screws, including four cortical screws and five locking screws. The surgical site was closed. No samples were submitted for culture at this stage. Postoperative CT confirmed correction within the reference range, with TC–CnT 4.7° and CdC–CnT −4.3° (Figure 2D).

At 4 weeks after deformity correction, a draining tract developed over the osteotomy site. Radiographs showed aggressive periosteal reaction and osteolysis, with progressive widening of the osteotomy gap on serial images (Figure 2E). Swab culture and susceptibility testing confirmed methicillin-resistant *Staphylococcus aureus* (MRSA), which was resistant to doxycycline but sensitive to gentamicin and vancomycin. Osteomyelitis and infected delayed union were suspected at the osteotomy site, with concern for progression toward non-union because early distinction between delayed union and non-union can be challenging and requires serial assessment.

### 2.4 Surgical management of infected delayed union and follow-up

**Figure 3:**
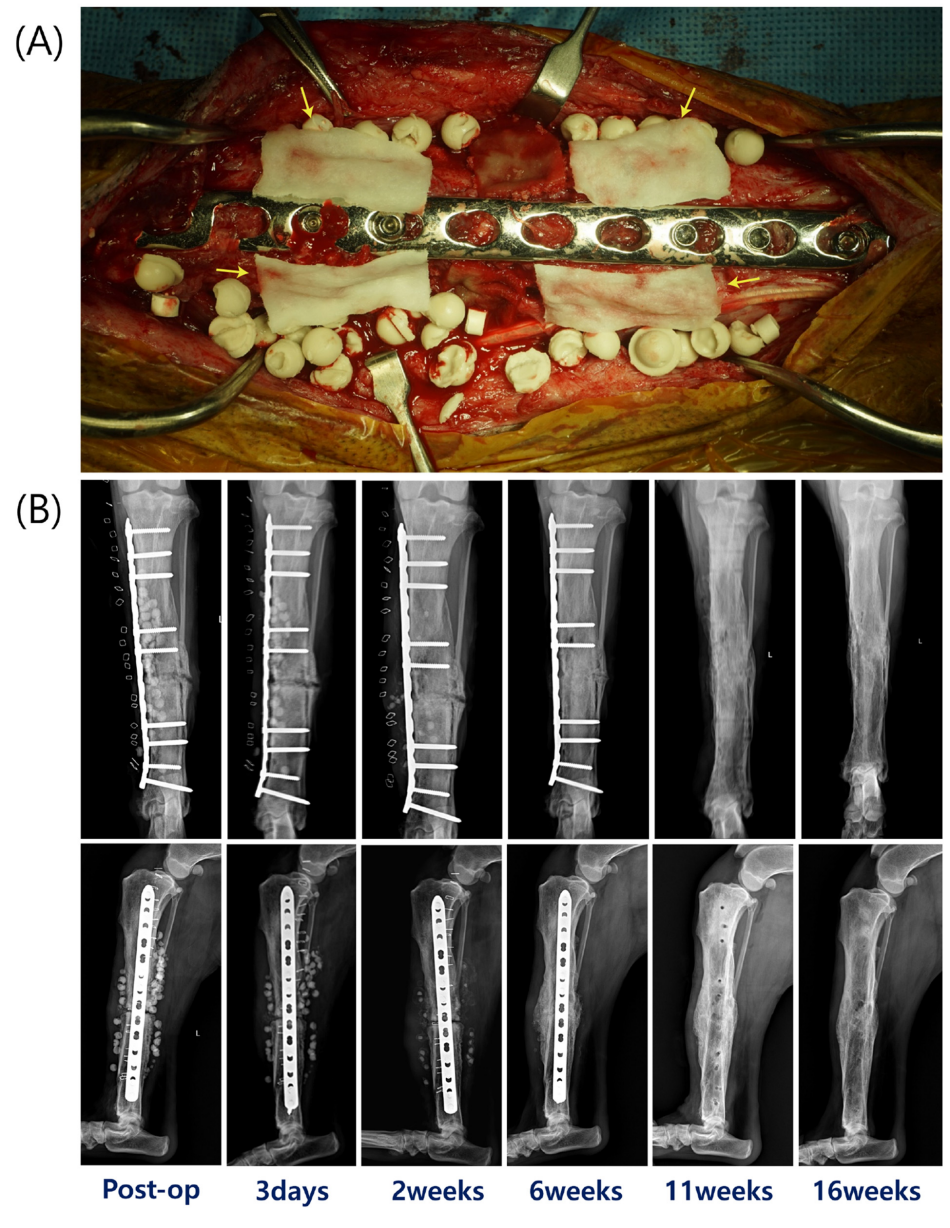
**(A)** The surgical grafting procedure using calcium sulfate antibiotic beads with HA, cancellous allograft chips, and rhBMP-2. Yellow arrows indicate the gentamicin-soaked collagen sponge. **(B)** Radiographs from left to right: immediately postoperatively, 3 days, 2, 6, 11, and 16 weeks postoperatively.

At 5 weeks after the second surgery, a third surgery was performed to implant antibiotic beads and bone graft materials. Under anesthesia, a medial skin incision was made over the tibia, and the subcutaneous tissue and fascia were dissected. Necrotic bone tissue around the fracture line was debrided until bleeding from viable bone was observed, and the surgical field was irrigated with sterile 0.9% saline solution. Calcium sulfate antibiotic beads (OsteoSet, Wright Medical Technology, TN, USA) were prepared by mixing 25 g of calcium sulfate powder with 320 mg of gentamicin and 1,000 mg of vancomycin. The mixture was poured into a bead tray to set, producing beads implanted at the surgical site. HA (Novosis Dent, CGBIO, Seoul, Republic of Korea) and an allograft (VTB cancellous chips; Veterinary Instrumentation, Sheffield, UK) were grafted together with 250 μg/ml rhBMP-2 (Novosis Dent, CGBIO, Seoul, Republic of Korea) to promote bone healing. Gentamicin-soaked collagen sponges were applied around the osteotomy site, covering the tibia both proximal and distal to the osteotomy, for local management of osteomyelitis (Figure 3A) (19, 20). Intraoperatively, necrotic bone and swab samples from the surgical site were submitted for bacterial culture and antimicrobial susceptibility testing, which confirmed osteomyelitis.

On postoperative day 3, fever and soft tissue swelling decreased. By 2 weeks postoperatively, periosteal callus formation with a solid but mildly irregular pattern was observed on radiographic examination (Figure 3B). Serial radiographs showed progressive biodegradation of the beads, which were no longer visible at 6 weeks. Radiographs at 6 weeks supported bone union with hard callus formation (Figure 3B). At 11 weeks, implant removal was performed to reduce the risk of stress shielding (Figure 3B). At 16 weeks, there was no clinical evidence of osteomyelitis and the lameness score was 1/5 (21). Radiographs confirmed complete union of the tibial fracture with tibiofibular synostosis and solid periosteal new bone formation, resulting in cortical thickening, at the previous fracture site (Figure 3B). At 20 months, telephone follow-up indicated no noticeable lameness or pain during limb manipulation, and the owner was satisfied with the outcome.

### 2.5 Objective gait analysis

Objective gait analysis was performed using a pressure-sensitive walkway (Tekscan, Norwood, MA, USA) to obtain kinetic data during the stance phase. After acclimatization, the dog was walked across the walkway on a loose leash. Valid trials were selected by excluding runs with acceleration or deceleration and incomplete paw contact. Data were collected at 16, 27, 28, 32, and 44 weeks after the first surgery (T0). Changes were analyzed using repeated-measures ANOVA with Šídák's multiple comparisons test. Symmetry index (SI) and weight distribution (WD) were calculated using equations described (22, 23):

$SI=\frac{x_{R}-x_{L}}{\frac{1}{2}(x_{R}+x_{L})}\times 100\;$, $WD\left(\%\right)=\frac{Limb\;VI\;or\;PVF}{Total\;limbs\;VI\;or\;PVF}\times \;100$

**Figure 4:**
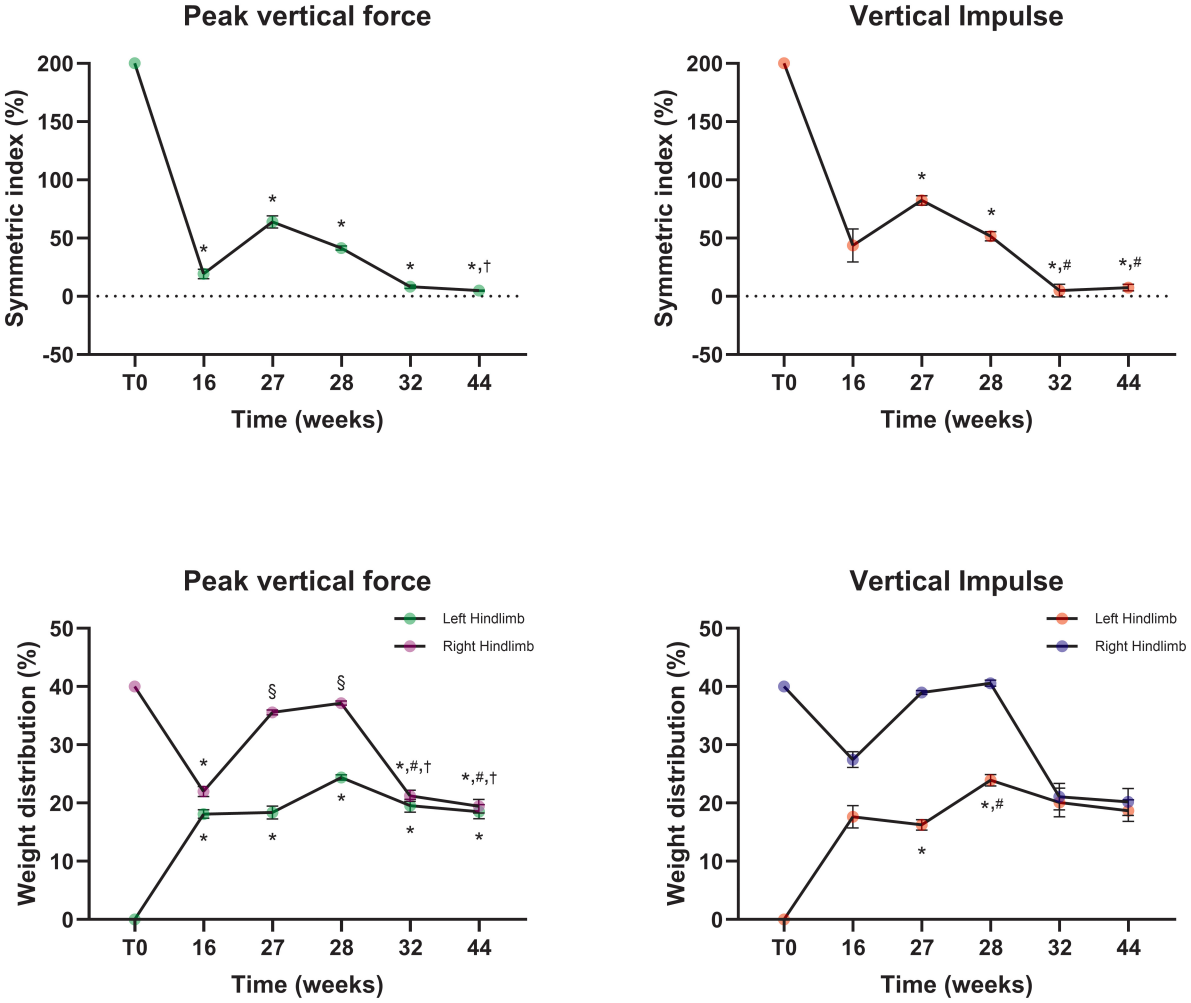
The graphs represent the symmetry index and weight distribution obtained from gait analysis over time. The first surgery (T0) corresponds to fracture stabilization. The gait analysis at 27 weeks after T0 (27 weeks) corresponds to 4 weeks after torsional deformity correction. The gait analysis at 28 weeks after T0 (28 weeks) corresponds to the immediate postoperative time point after implantation of calcium sulfate antibiotic beads and bone graft substitutes containing rhBMP-2. Statistical significance was indicated when the *p*-value was less than 0.0001. * indicates a significant difference from Pre-op. ^§^ indicates a significant difference from 1st-OP 16wks. ^#^ indicates a significant difference from 2nd-OP 4 weeks.^†^ indicates a significant difference from 3rd-OP.

At 16 weeks after T0 (lameness score of 2/5, development of a left tibial torsional deformity), the SI for peak vertical force (PVF) and vertical impulse (VI) indicated that SI-PVF was significantly lower than the T0 value (*p* < 0.0001) and that SI-VI was significantly reduced compared with the T0 value (*p* < 0.05), while WD-PVF and WD-VI converged toward 20%. At 27 weeks after T0 (lameness score of 4/5, when osteomyelitis and delayed union were suspected), SI-PVF and SI-VI increased relative to the 16-week values, and WD-PVF and WD-VI deviated from 20%. Between 28 and 44 weeks after T0 (lameness score of 1/5 at 32 weeks, with progressive bone union), SI-PVF and SI-VI decreased, and WD-PVF and WD-VI approached 20% by 32 weeks, with improvements maintained through 44 weeks (Figure 4).

## 3 Discussion

In this study, we used a plate–rod construct to achieve biological osteosynthesis in a complex tibial fracture, additionally supported by a type I ESF. Monocortical locking screws were used when bicortical purchase was not feasible. Prior studies support the biomechanical and clinical stability of monocortical screws in plate–rod constructs (24).

At 16 weeks after the first surgery, the radiographic appearance was most compatible with hypertrophic non-union. This configuration suggests biologically active repair with inadequate stability and persistent interfragmentary motion. Because internal fixation provided the primary mechanical stability across the proximal fracture region, the ESF was unlikely to add meaningful support, and prolonged retention could increase the risk of pin-tract infection and aftercare burden, so the ESF was removed. Osteolytic change was evident on the 16-week radiographs, and occult infection could not be excluded despite radiographic union at 20 weeks postoperatively. The possibility of occult infection may have been relevant to the infected delayed union that developed after the subsequent torsional deformity correction. Culture positivity and microbial colonization of orthopedic implants have been reported despite uncomplicated healing, with 20/49 explants (40.8%) classified as infected in one study (25). These findings highlight that occult infection cannot be excluded even with apparently progressive radiographic healing.

Accurate intraoperative assessment of tibial torsional alignment remains limited, and visual inspection and fluoroscopic comparison with the contralateral limb can be difficult and unreliable (17). In this case, fracture complexity and reliance on visual assessment constrained the ability to achieve and verify torsional alignment during the initial fixation. Consequently, a clinically relevant tibial torsional deformity was recognized, necessitating corrective surgery. The timing of onset could not be determined because early postoperative radiographs were not consistently obtained in a standardized position suitable for tibial torsion assessment, and torsional malalignment before ESF removal may have been underestimated or not recognized. In dogs, tibial torsional deformity has not been systematically reported as a distinct outcome after tibial fracture fixation; for example, malunion was reported in 0.7% of 461 canine fractures without stratification by deformity plane (2). By contrast, human CT studies have documented clinically relevant postoperative tibial torsion (26). Collectively, these data support CT-based three-dimensional quantification and additive manufacturing as tools to improve preoperative planning in complex cases, and patient-specific 3D-printed constructs have been proposed as one practical approach for small-animal orthopedics (27).

Despite sterile technique, postoperative osteomyelitis was likely multifactorial. The patient underwent multiple surgeries, and repeated operations are a risk factor for osteosynthesis-associated infection (28). Outdoor housing and reduced owner compliance may have compromised postoperative wound care and increased infection risk. A single-stage strategy was selected to treat osteomyelitis and promote healing using internal fixation, local antibiotic therapy, and bone regeneration. The use of antibiotic-impregnated calcium sulfate beads in a single surgical episode has been reported in dogs with deep orthopedic surgical site infections (8). In that retrospective case series, infection resolution was achieved in 6 of 10 cases and was more frequent when the bead antibiotic matched bacterial susceptibility. In the present case, local antibiotic selection was guided by culture and susceptibility testing and was incorporated into a single-stage approach integrating definitive stabilization and bone regeneration (8).

Given gentamicin's concentration-dependent bactericidal activity, achieving a high early peak exposure is pharmacodynamically desirable. Sustained local exposure may help suppress residual bacteria and limit biofilm formation. Consistent with this rationale, previous studies report that gentamicin-impregnated collagen sponges release gentamicin rapidly, reaching peak concentrations within 0.5–1.0 h and then declining steeply within 24 h (29). By contrast, calcium sulfate antibiotic beads can elute antibiotics for up to 28 days (30) and reduce bacterial colonization and biofilm formation (31). In a clinical series of small animals with deep orthopedic surgical site infections, calcium sulfate antibiotic-impregnated beads were successfully used as an adjunct to surgical debridement, supporting their clinical applicability for local infection control (8). Thus, concurrent use of both carriers can be viewed as a complementary strategy coupling early peak exposure with prolonged local antibiotic release. This approach requires predictable calcium sulfate resorption even with concurrent rhBMP-2–loaded graft materials. In our case, the radiographic disappearance of calcium sulfate occurred within the resorption period reported in prior clinical studies (6). This provides indirect evidence that concurrent rhBMP-2–loaded graft materials did not impede calcium sulfate biodegradation.

In this case, HA was combined with cancellous allografts to provide osteoconductive scaffolding and maintain structural stability during healing, and rhBMP-2 was incorporated to augment osteoinduction. These choices are supported by prior reports indicating that allografts may resorb more rapidly when used alone, potentially shortening the duration of structural support (32). In addition, previous studies have noted that cancellous allografts are only weakly osteoinductive and that HA is non-osteoinductive (32, 33). Taken together, this rhBMP-2–loaded cancellous allograft and HA composite can be viewed as a bone tissue engineering approach that integrates osteoinductive signaling with osteoconductive scaffolding and structural support. To contextualize this course, time to radiographic union was interpreted relative to prior reports. Previous studies indicate that uncomplicated long-bone fractures typically achieve radiographic union within 6–8 weeks, whereas cases complicated by delayed union or osteomyelitis often require substantially longer (2). In this context, after local antibiotic delivery combined with a bone tissue engineering approach, radiographic union was confirmed at 6 weeks. This comparison suggests that the combined strategy may support earlier bone union in infected delayed union.

Regarding rhBMP-2 dosing, prior studies reported improved healing with 200 μg/ml delivered via an absorbable collagen sponge in a 1 mm tibial defect gap (20). In our patient, the tibial defect gap measured 1.4 mm, which exceeded the 1 mm gaps described in that report. Accordingly, a rhBMP-2 concentration of 250 μg/ml was selected, supported by a prior clinical report describing functional recovery of long-bone non-union with 250 μg/ml of rhBMP-2 (34). In this case, infected delayed union resolved with radiographic union, supporting feasibility of this dosing approach. Notably, there are no established guidelines for BMP dosing tailored to specific bone defect conditions in dogs, and the selected dose in this case was extrapolated from the available clinical evidence and the defect size, while the optimal rhBMP-2 dose for comparable defects remains to be determined. In addition, the concurrent use of cancellous allograft may have increased the early release of rhBMP-2, potentially contributing to thick periosteal new bone formation at the mid-tibial diaphysis after the third surgery.

At late follow-up, kinetic indices suggested restoration of hindlimb loading symmetry. Specifically, SI-PVF values were 8.23 at 32 weeks and 4.81 at 44 weeks after T0, and both were below the SI < 9 threshold that has been used as a reference indicator of relatively normal gait symmetry in postoperative dogs (35). In a previous report, an SI-PVF value of 31.97 was reported at 4 weeks after TPLO, with improvement over subsequent months (36). Compared with this recovery trajectory, the magnitude and timing of symmetry restoration observed in this infected delayed union case appear clinically favorable. Additionally, left pelvic-limb WD-PVF values were 19.56 and 18.52% at 32 and 44 weeks, respectively, indicating near-balanced hindlimb weight distribution. Nonetheless, SI and WD values can be influenced by gait velocity, acceleration, and trial selection (37). Therefore, SI and WD should be interpreted cautiously, with emphasis on standardized acquisition conditions and within-dog longitudinal trends (38). These findings suggest that regenerative treatment combined with calcium sulfate antibiotic beads may have contributed to restoring gait symmetry that had been reduced by infected delayed union.

This report is limited by its single-case report, which restricts generalizability and precludes comparison with alternative strategies. In addition, the higher cost of rhBMP-2 is a practical limitation, although it may be cost-effective in complex cases at high risk of non-union or revision surgery (39). Further studies are needed to clarify optimal antibiotic and growth factor dosing and to better define interactions among calcium sulfate beads, HA, allografts, and rhBMP-2 during bone regeneration.

## 4 Conclusions

This case report describes the successful treatment of infected tibial delayed union in a dog with a history of multiple surgeries. By combining vancomycin- and gentamicin-loaded calcium sulfate antibiotic beads with bone graft substitutes containing hydroxyapatite, allograft, and rhBMP-2, we achieved resolution of infected delayed union with restoration of gait function, confirmed by serial radiography and objective gait analysis. This case highlights the value of integrating local antibiotic delivery with osteoconductive and osteoinductive graft materials in infected delayed union. However, further studies are needed to define optimal dosing, material combinations, and indications for these biomaterials.

## Data Availability

The original contributions presented in the study are included in the article/supplementary material, further inquiries can be directed to the corresponding author.
